# Reducing health inequities: the contribution of core public health services in BC

**DOI:** 10.1186/1471-2458-13-550

**Published:** 2013-06-06

**Authors:** Bernadette (Bernie) Pauly, Marjorie MacDonald, Trevor Hancock, Wanda Martin, Kathleen Perkin

**Affiliations:** 1 School of Nursing and, Centre for Addictions Research of BC (CARBC), University of Victoria, Box 1700 STN CSC, Victoria, BC V8W 2Y2, Canada; 2 School of Nursing, University of Victoria, Box 1700 STN CSC, Victoria, BC V8W 2Y2, Canada; 3 School of Public Health and Social Policy, University of Victoria, Box 1700 STN CSC, Victoria, BC V8W 2Y2, Canada; 4 Centre for Addictions Research of BC, University of Victoria, Box 1700 STN CSC, Victoria, BC V8W 2Y2, Canada

**Keywords:** Health inequities, Health equity tools and frameworks, Public health services, Population health interventions, Public health policy, Determinants of health, Ethics and values, Complex adaptive systems, Intersectionality, Critical social justice

## Abstract

**Background:**

Within Canada, many public health leaders have long identified the importance of improving the health of all Canadians especially those who face social and economic disadvantages. Future improvements in population health will be achieved by promoting health equity through action on the social determinants of health. Many Canadian documents, endorsed by government and public health leaders, describe commitments to improving overall health and promoting health equity. Public health has an important role to play in strengthening action on the social determinants and promoting health equity. Currently, public health services in British Columbia are being reorganized and there is a unique opportunity to study the application of an equity lens in public health and the contribution of public health to reducing health inequities. Where applicable, we have chosen mental health promotion, prevention of mental disorders and harms of substance use as exemplars within which to examine specific application of an equity lens.

**Methods/design:**

This research protocol is informed by three theoretical perspectives: complex adaptive systems, critical social justice, and intersectionality. In this program of research, there are four inter-related research projects with an emphasis on both integrated and end of grant knowledge translation. Within an overarching collaborative and participatory approach to research, we use a multiple comparative case study research design and are incorporating multiple methods such as discourse analysis, situational analysis, social network analysis, concept mapping and grounded theory.

**Discussion:**

An important aim of this work is to help ensure a strong public health system that supports public health providers to have the knowledge, skills, tools and resources to undertake the promotion of health equity. This research will contribute to increasing the effectiveness and contributions of public health in reducing unfair and inequitable differences in health among population groups. As a collaborative effort between public health practitioners/decision makers and university researchers, this research will provide important understanding and insights about the implementation of the changes in public health with a specific focus on health equity, the promotion of mental health and the prevention of harms of substance use.

## Background

It is an unfortunate feature of Canadian society that not everyone enjoys equal opportunity for good health. Despite the existence of universal health care in Canada, poor health is disproportionately borne by those who are disadvantaged in relation to factors such as socioeconomic status, geographic location, gender and ethnicity. For example, there are significant differences in life expectancy among geographic regions; those with low incomes have significantly poorer health than those with high incomes, and decreased life expectancies and poorer health exist among Aboriginal peoples compared to the general population [1,2]. These disparities in health, known as health inequities, are potentially avoidable, remediable and a consequence of structural injustices that shape social conditions in ways that disadvantage some groups in the population [3,4].

Reducing health inequities within and between countries is an ethical, social, and economic imperative and a goal of health systems worldwide [3,5,6]. Health equity has been a priority for the Canadian public health sector since the release of the World Health Organization’s Ottawa Charter for Health Promotion in 1986 [1,2,7-10]. In 2008, Canada’s Chief Public Health Officer, Dr. David Butler Jones, clearly identified health inequities reduction as a priority for the country and for public health [2]. Public health does not have sole responsibility for promoting health equity, but public health does have a critical role in reducing health inequities [1,9,11,12]. Several Canadian reports have recommended strengthening public health infrastructures, supporting effective delivery of public health services, and increasing collaboration both within the health care system and with other sectors to achieve common health goals, including health equity [13-17]. Specifically, public health can contribute to reducing health inequities by integrating health equity considerations into policy and programs, collaborating with other sectors to address inequities, engaging with communities to support their efforts to address inequities, identifying the reduction of health inequities as a strategic priority, and strengthening knowledge development and exchange around issues related to health equity [1].

In British Columbia (BC) Canada, the site of this study, public health renewal efforts have incorporated application of an equity lens [18]. In this instance, an equity lens is a way of approaching public health policy and program development that takes into account disadvantages suffered by some people because of their social positioning. In BC, there are substantial health inequities between regions and among different groups in the population [11,19]. BC has one of the highest poverty rates in Canada and the highest child poverty rate [11]. Among those living in poverty, there is a higher concentration of chronic illness and poor mental health as well as unmet health care needs and difficulties accessing health care [11]. Health behaviours such as smoking, low physical activity, and poor diet with accompanying obesity are more prevalent in low income groups and a reflection of socioeconomic conditions. Among those most affected by health inequities in BC are Aboriginal peoples, the working poor, people with mental illness and addictions, new immigrants, and those impacted by homelessness. The use of an equity lens is meant to draw attention to these disadvantages and encourage strategies to promote health equity in BC.

Recently, BC has experienced a process of public health renewal. Beginning in 2005, a Framework for Core Functions in Public Health was created to guide public health renewal [12,20]. The framework identified the public health services and supports that BC’s health authorities are expected to provide. In addition to public health priorities, the framework describes two lenses that should be applied in public health programs to address health inequities and ensure that the health needs of particular groups and the overall population are met: a population and an equity lens. This research is concerned with the integration and application of the equity lens in public health. The BC Core Functions Framework [12,20] is unique in Canada. Three innovative aspects of the core functions implementation in BC are that: 1) it is a major policy level population health intervention; 2) the planned process constitutes a ‘natural experiment’; and 3) the process of implementation is an integrated and evolving knowledge translation and exchange (KTE) process. Its launch created a unique opportunity to study the implementation and impact of public health renewal. Public health knowledge users have identified that health equity is often not a priority in health systems and that application of an equity lens is often challenging with little practical guidance available. Thus, health equity was identified as a dominant research priority for both researchers and knowledge users [21].

It is too unwieldy to study the application of an equity lens within the implementation of the core functions framework in its entirety. Thus, we are using the two program areas *mental health promotion/prevention of mental disorders* and *preventing the harms of substance use* as ‘exemplars’ to explore our specific research questions related to reducing health inequities in BC. Since the Core Functions Framework was implemented in BC, there have been two significant policy developments: BC’s ten-year plan for mental health and substance use, *Healthy Minds, Healthy People*[22], and the adoption of Key Result Areas as the Ministry of Health’s way of communicating outcome expectations to the health authorities. The key areas of service delivery focus on promoting and improving the overall health of the population, and addressing the needs of specific groups [23]. The Core Functions Framework and *Healthy Minds, Healthy People* provide a focus for exploring the potential to reduce health inequities through public health policy and services in BC.

Both the Canadian Institutes of Health Research (CIHR) and the Population Health Intervention Research Initiative of Canada (PHIRIC) define population health interventions [24,25] as “…policy and program interventions that operate within or outside the health sector and have the potential to impact health at the population level.” [26] (p. I5). Population health interventions (PHIs) often work through the implementation of public health services. Clearly, the Core Functions Framework meets this definition. Coupled with *Healthy Minds, Healthy People (HMHP)*[22], these two policy interventions provide a focus for exploring the potential to reduce health inequities through public health policy and services in BC.

Cameron and Riley [27] argue that some of the most relevant population intervention studies “involve learning from evaluating ‘natural experiments’ as innovative policies or programs are introduced” (p. 5) and that these studies “enable us to ‘learn as we go’ about what works, with whom, and under what circumstances”. The recent processes of public health renewal in BC provide such a “natural experiment”. The design of this study allows for comparisons between early phases of implementation and later ones with regard to the inclusion and conceptualization of health equity in public health. This research also allows for comparisons between health authorities. Each health authority is taking up the Core Functions Framework and undertaking strategies for mental health promotion and preventing the harms substance use in unique ways that fit their own context.

The purpose of this program of research is to study and foster learning about the use of an equity lens during a period of complex system change in public health to inform systemic responses for reducing health inequities. The goals of this research are to:

1. Identify and understand the contextual influences that promote uptake of health equity as a priority in the health system and determine the extent to which health inequities are a priority for health systems in general and in relation to the promotion of mental health, prevention of mental disorders and harms of substance use;

2. Explore and examine the engagement of public health with other sectors in health inequities reduction in the areas of mental health promotion and prevention of harms of substance use;

3. Critically analyze the theoretical and practical utility of existing equity tools for public health and inform program development, learning and capabilities requirements to apply relevant tools; and

4. Develop a theoretical understanding of the ethical issues encountered by public health practitioners in their efforts to reduce health inequities and the process of managing those tensions.

5. Engage in an innovative knowledge translation and exchange (KTE) process to strengthen and improve health sector innovation for reducing health inequities.

## Methods/design

This research is made up of four related projects, each investigating health equity in BC’s public health policies and programs:

1. Assessing health equity priorities and strategies

2. Intersectoral action in public health

3. Theoretical and practical relevance of equity tools

4. Power and ethics in public health

Each of BC’s health authorities constitutes a case in this research. In BC, there are five regional health authorities and one provincial health services authority. Since the study was funded, an interim First Nations Health Authority has been created but is early in its development. Currently, the one provincial and five regional health authorities are partners in the research and each has public health representatives on the research team. The Ministry of Health has a stewardship role in the implementation of the Core Functions Framework and BC’s HMHP providing direction and support to the health authorities, monitoring and evaluating public health services, and intervening strategically when needed. The regional health authorities are responsible for the delivery and quality management of public health services within their jurisdiction, including mental health promotion, preventing mental disorders and harms of substance use. In BC, public health programs may be provided by a range of service areas within the health authority and not only by traditional providers of public health services. Within the Core Functions Framework, several core programs require collaboration within and beyond the health care system. Thus, we have a unique opportunity to study differences among the cases in how health equity is incorporated in policy and programs, not only by public health but also across the health authorities and in collaboration with other sectors (e.g., housing, social services and education).

In this research, we use a participatory process in which knowledge users and academic researchers work collaboratively throughout the entire research process from developing the research questions, through planning the research design, to analyzing and interpreting the data. Participatory Action Research (PAR) seeks to democratize the research process and acknowledges the role of communities in knowledge generation [28]. PAR fosters learning through reflexive and dialectical social processes and aims to reform theory/knowledge and practice so that they serve each other [29]. Participatory approaches are aligned with the process of integrated knowledge translation and exchange.

This research has ethical approval from the University of Victoria Human Research Ethics Board (protocol number J2011-102). The board follows the *Tri-Council Policy Statement for Research Involving Humans*[30], which is consistent with the *World Medical Association Declaration of Helsinki – Ethical Principles for Medical Research Involving Human Subjects*[31]. Ethical approval has been also received from the six participating regional health authority research ethics boards.

### Study 1: Assessing health equity priorities and strategies

The purpose of our first study is to assess health equity priorities and strategies in the health authorities. We will first conduct a baseline assessment and comparative analysis across health authorities of the status of health equity initiatives and, second, a follow up to assess change over time. The intent is to “scan” the cases to determine current activity on health equity and inequity reduction in general in each health authority and what may be happening with particular respect to mental health promotion and prevention of harms of substance use. We will also look at whether and how health equity issues have been prioritized, the contextual influences on priorities and health equity plans/strategies, and how and what explains these changes over the course of the study. The result will be six unique case reports for each period of data collection (baseline and follow-up), an assessment of changes over time within each case, a cross case comparison at baseline and follow-up, and an overall provincial level analysis that summarizes across cases and times. Knowledge translation and exchange activities will feed these results back to each individual health authority, and to the entire team. The specific research questions that we seek to answer in Study 1 are:

1. To what extent has health equity, in general, been identified and prioritized across the health authorities as reflected in core health authority documents and plans?

2. What are the contextual influences on priority setting and equity goals at the organizational systems level?

3. What specific strategies are proposed and implemented by public health to reduce health inequities through mental health promotion and prevention of harms of substance use? How has the provincial, regional and community context influenced the selected mental health and substance use health equity strategies and what is the impact of the context on the development, implementation and outcome of these strategies?

4. What are the changes with respect to the above over time? (Comparative case analysis from baseline to follow-up)

These questions will be answered using a range of methods including documentary review and semi-structured qualitative interviews in each health authority. We are drawing on a grounded theory approach and will analyze the data using content, critical discourse and situational analysis.

### Document review and analysis

Documents are important to the work of governments and organizations as essential tools that guide thinking and action in relation to establishing authority and processes as well as framing the problem, making recommendations and tracking implementation [32]. “In each case, the progression or movement of knowledge into action and research into policy is channeled through a document, a signal expression of findings and recommendations that constitutes a critical moment or node in a complex network of processes and relationships” [32] (p. 52). At baseline and follow up, we will undertake a review and analysis of provincial and health authority documents that outline goals, vision, mandates and strategic directions. These might include strategic plans, service plans, health equity plans and health authority reports to government for accountability purposes. Our partners in government and health authorities will assist us in gathering a complete collection of documents.

The documents will be analyzed using content and critical discourse analysis. Initially, using NVivo 9.0, we will examine and code the documents for content related to priorities and health equity. We will use critical discourse analysis to illuminate dominant values, discourses and discursive dynamics that influence prioritizing (or not) of health inequities and strategies for reducing health inequities. Our intent is to make explicit how language itself, in concert with other structures and processes of communication, reflects and reinforces power in social processes. Incorporating analytic techniques described by Van Dijk [33], we will consider how text and patterns of communication may serve to produce or sustain health equity as a priority and how it is understood and taken up within each health authority at baseline and at follow up two years later. Document analysis can be a way to understand both the extent to which health equity is a priority and the way it is acted upon by tracking and documenting implementation of initiatives over time [32].

### Individual in-depth interviews

In addition to the contextual influences on priorities that will be identified in the document review, we will explore the context in which health inequities reduction is undertaken through in-depth interviews with key people in senior management roles in each of the six health authorities. The specific interview questions will be informed by the findings of the document review and further refined in collaboration with the research team.

### Data analysis

We will use the constant comparative method of grounded theory for line by line coding and categorization of the data, to contribute to situational analysis. In Study 1, our intent is not to generate a final grounded theory, but to allow sufficient open ended and higher level coding to permit a situational analysis, which is based on the analytic processes of grounded theory. Grounded theory was developed by Glaser and Strauss [34] for the purpose of creating theoretical explanations of basic social and structural processes. Grounded theory’s constant comparative method has since been elaborated by Miles and Huberman [35] for multi-site case study data analysis. What makes this analytic method particularly relevant in this study is its grounding in ecological principles [36,37] that are consistent with complex adaptive systems thinking [38]. For this study, rather than a full grounded theory, our analytic products will be a series of situational maps.

Situational analysis [39] provides techniques to analyze and map context and its influence on the complexities of situated interaction. In this approach, the emphasis shifts from the basic social process in grounded theory to the situation in which the process takes place. In keeping with a complex adaptive systems approach, situational analysis directs our attention to the ongoing dynamics of the context and the interactions and consequences of interventions [40] inserted as “events in systems” [41]. In situational analysis, three types of maps are produced [39]: situational maps that lay out the major human, non-human and discursive elements in the situation of inquiry; social world arenas maps that lay out the collective actors, key non-human elements and the “arenas of commitment”; and positional maps that lay out the major positions taken vis-à-vis particular axes of difference, concern and controversy around issues in the situation of inquiry. This analysis will also incorporate the findings of the document review of priorities and strategies related to health inequities reduction; discourse analysis is, in fact, integral to situational analysis.

### Cross case analysis

Cross case analysis will be done using techniques proposed by Miles and Huberman [35] for data display to aid interpretation, which support a systematic analysis of commonalities and differences within and across cases. We will produce relational networks, as well as time-ordered and conceptually ordered data displays within and across cases. Once our individual case reports are produced, we will do a comparative analysis within cases to determine changes from baseline to follow-up, and will complete the process by producing an overall provincial level report that summarizes the findings across cases and times.

### Study 2. Intersectoral collaboration for health inequities reduction

The purpose of the second study is to explore the extent and nature of relationships and collaborations between public health and others within and outside the health authorities with respect to reducing health inequities through the promotion of mental health and prevention of mental disorders and harms of substance use. Our research questions are:

1. Who do public health practitioners engage with inside of the health authority on health equity issues related to mental health promotion and prevention of mental disorders and harms of substance use?

2. Who does public health engage with outside of the health authority on health equity issues related to mental health promotion and prevention of mental disorders and harms of substance use?

3. Who are prominent actors/organizations in social networks for promotion of health equity?

4. What opportunities exist to strengthen intersectoral engagement in the promotion of health equity in programs related to the promotion of mental health and prevention of mental disorders and harms of substance use?

5. How does this change over time?

All Study 2 research questions will be addressed through two social network analyses: one at baseline and one at follow-up. These two analyses will be compared to look for any changes in patterns of collaboration. Social network analysis is a useful strategy to build capacity for strengthening collaborations [42] so the results may help improve intersectoral action for reducing health inequities. Social network analysis is a well-established methodology, emerging from sociology and anthropology, for collecting and analyzing data from multiple individuals or organizations that may be interacting with one another, and describing these relationships [43-47]. Social network analysis provides information on social structures through a study of networks and the position of social actors within the social context. Social network analysis has been used in a variety of applications related to health care delivery and heath policy but only recently in public health applications [48].

### Sampling and data collection

With the help of our health authority partners, we will generate an initial short list of key focal actors who work in the health authority’s public health mental health promotion and prevention of harms of substance use programs/services and proceed from there to begin snowball sampling. Health authority partners will be actively involved in working with the researchers in defining the sampling plan and the questionnaire because of their greater familiarity with health authority structure and the nature of collaboration within and outside its boundaries. We will pilot test both the sampling strategy and the questionnaire with a small sample in one health authority. For social network analysis to be done effectively, a response rate of 85-90% is essential. Face to face interviews are generally the most effective in producing the highest response rate [49]; online surveys are less effective. As face to face interviews are both expensive and impractical, we propose doing telephone interviews with strong reminder and follow up [49] to achieve the necessary response rate. Our strong working relationships with the health authorities and our prior research with them give us credibility that should enhance the response rate.

### Data analysis

Social network analysis produces visual representations of the number and type of relationships between network actors. At the individual level, measures of connectivity (degree to which individuals are linked), centrality (importance or prominence of actors expressed through measures of betweenness, closeness, degree and prestige) and structural equivalence will be used. This can help identify people who play central roles in the promotion of health equity. We anticipate that social network analysis will facilitate learning about intersectoral collaboration as a key role of public health in the promotion of health equity.

### Study 3: Assessing the theoretical relevance and practical utility of health equity tools

The Core Functions Framework indicates that an equity lens should be applied in all public health programs and strategies. However, there is no clear guidance for health authorities on how to apply the lens in their policies and programs. In two of our previous studies, we found that decision makers and practitioners understand and apply equity in very diverse ways and overall are challenged in doing this work. The purpose of this study, therefore, is to identify and analyze existing health equity tools and to determine their relevance, both theoretically (to ensure that they adequately incorporate equity and social justice considerations) and practically (to ensure their utility and relevance to practitioners). Research questions guiding this study are:

1. What health equity tools are available?

2. What is the theoretical relevance of available tools?

3. What is the practical utility of available tools for guiding decision makers and practitioners in developing, implementing and evaluating polices, programs/services aimed at reducing health inequities in general and with specific application to promoting mental health and preventing harms of substance use?

This study builds on work undertaken by the Public Health Agency of Canada (PHAC) to review equity-focused health impact assessment tools and resources and create a comprehensive summary of these tools [50]. However, the theoretical relevance and practical utility of these health equity tools has not been assessed for use of the tools in local or national contexts or for application to specific programs such as mental health promotion, prevention of mental disorders and harms of substance use.

### Creating an inventory

The PHAC work identified a range of health equity impact assessment and other health equity tools. Our purpose is to extend the original PHAC scan by identifying a comprehensive set of equity tools, developed for use in public health. First, we will identify all published guides, frameworks and toolkits that have an explicit focus on enhancing health equity. Second, we will undertake a search of peer-reviewed published literature. Third, relevant journals such as International Journal for Health Equity will be hand searched. Standard procedures for reference tracing and follow up will be used to generate a comprehensive inventory of the published literature. Fourth, we will ask research team members to identify health equity resources they are aware of and will work closely with PHAC and the Canadian National Collaborating Centre on Determinants of Health to identify and contact experts in the field who may be aware of additional tools and resources.

### Assessing theoretical relevance

Each of the tools that meet the initial screening criteria above will be included in an assessment of theoretical relevance. An initial assessment of the health equity tools’ theoretical orientations will be conducted. Key considerations included attention to 1) structural determinants of health outcomes; 2) attention to social, political, historical and cultural roots of social disadvantage; and 3) distribution of power in both the production of inequities and policy processes. A review guide outlining important theoretical criteria will be developed. This assessment will identify the theoretical underpinnings of identified tools. Health equity tools that have theoretical relevance will next be assessed for practical utility using criteria developed through a concept mapping exercise with public health practitioners.

### Developing criteria for and assessment of practical utility

To develop criteria to assess the practical utility of equity tools for use in public health programs, we will use concept mapping [51-53]. This is a structured conceptualization process that can be used by groups to develop a conceptual framework to guide evaluation and planning. Concept mapping can be used to conceptualize goals, needs, resources, capabilities, strategies or other dimensions of a plan. This method enables groups to describe and generate ideas in response to a focused question. The ideas can be represented in the form of a visual map through use of multivariate statistical techniques. It is a method specifically geared for systems thinking and fits well within our complex adaptive systems framework and our participatory approach [54]. Concept mapping, as developed by Trochim, consists of series of steps [51].

Step 1 involves determination of who will participate in the process and then engages participants in development of the focused questions [51]. Participants for this initial phase will be those engaged in developing and implementing health authority health equity plans and mental health promotion, preventing mental disorders and harms of substance use programs in public health. Trochim indicates that the process of concept mapping is enhanced when a wide variety of relevant people with multiple viewpoints are involved. He recommends 10–20 people but up to 75 people is possible at this stage. Not all participants need to be involved in every stage. The focused questions act as a prompt for Step 2: the generation of concepts for mapping. A proposed prompt is “The important elements of an equity lens are…..”. Concept Systems software will be used for analysis. At this phase, decisions are also made about the rating scale and criteria, such as importance and feasibility, on which the responses will be rated [51].

In Step 2, we will invite public health practitioners to respond to the focused prompt in a virtual brainstorming session [51]. This phase will be completed over the internet, on a secure site. An advantage of this approach is that all participants can see what the others have entered and there is an opportunity for one person to spark another’s ideas [51]. Participants can generate an unlimited number of statements but Trochim recommends a total of no more than 100 statements to avoid serious practical constraints. If participants generate more responses, Trochim recommends either randomly selecting 100 statements or collapsing statements that are similar in nature by doing a thematic analysis. Step 3 begins when the statements are distilled and clarified so all the participants can understand the essential meaning. In this phase, we will ask participants to log on to the Concept Systems website where they will be able to sort the statements into themes or categories based on the similarity of ideas [51]. They also rank the statements on a Likert-type scale, according to the dimensions chosen in Step 1. In this study, participants will be asked how important and how feasible each idea is for judging the practical utility of an equity tool. Additionally, we will ask demographic questions to allow for sub-group analysis such as separating the responses of managers from front-line practitioners.

Step 4 involves statistical analysis and production of concept maps [51]. The program will create a similarity matrix based on the clustering of similar statements. The total similarity matrix is analyzed using non-metric multidimensional scaling (MDS) on two dimensions, allowing for representation on an XY axis that is called a point map. The point map output is fed into hierarchical cluster analysis that partitions the configuration into non-overlapping clusters (called a cluster map). Starting with about 15 clusters, we will examine each group of statements to make sense of the grouping. Additionally, the importance and feasibility ratings are averaged across participants for each item and each cluster. This produces a point-rating map. A point-rating map can be produced for the entire set of statements and for each cluster and shows average rating for each statement and cluster respectively.

Step 5 is the interpretation of maps [51]. This process involves careful reading of the statements, assigning descriptive names and higher level conceptual ordering [51]. Health authority partners will participate in the interpretive process. Based on the clusters, the team will develop a set of criteria for examining the practical relevance of the equity tools to the “real world” situations faced by practitioners. Thus, the outcomes of the concept mapping process will be the criteria necessary to evaluate practical relevance of the tools. We will use this to identify a list of health equity tools that meet the criteria. Once these are identified, health authorities can identify the capacity development requirements for widespread use of the health equity tools in practice.

### Study 4: power and ethics in public health

Potential ethical concerns abound in the development of public health programs and services to reduce health inequities. These processes can be politically charged requiring engagement across power structures and development of trust with communities most affected by inequities. Particular ethical concerns exist in relation to implementation of universal and targeted public health programs. In this study, the research questions are:

1. What are the specific ethical issues encountered by public health practitioners in their efforts to reduce health inequities in relation to mental health promotion, prevention of mental disorders, and prevention of harms of substance use?

2. How do public health practitioners navigate and manage these in their practice?

3. What insights can be generated for development of theoretical frameworks and public health ethics resources?

In this fourth study, building on studies 1 and 3, our purpose is, first, to explore the specific ethical concerns in practice related to reducing health inequities through mental health promotion, preventing mental disorders and harms of substance use programs from the perspective of people delivering programs. Second, we propose to construct a theoretical understanding of the processes by which decision makers and practitioners confront, negotiate, engage with and manage these ethical concerns. As with the first three studies, Study 4 will be conducted within a multiple case study design, to allow within-case analysis and cross-case comparisons, relying primarily on qualitative data from interviews.

As in Study 1, the constant comparative method of grounded theory will support situational analysis and the production of a range of visual maps relevant to each case. Unlike Study 1, however, the final analytic product will be a grounded theory that synthesizes across cases to describe, theorize, and explain the management of ethical challenges faced by practitioners as they engage in their day to day practice. Based on this analysis, we will develop a draft ethical framework for public/population health programs focused on reducing health inequities. This will be taken back to our practice partners for discussion and assessment of practical relevance, then revised and finalized for broader assessment and testing.

### Sampling and data collection

Grounded theory requires a purposive sampling strategy in which interview participants must have experience with the phenomenon under investigation. With assistance from our health authority partners, we will identify approximately ten public health practitioners in each health authority who work directly in providing mental health promotion and prevention of harms of substance use services/programs. In grounded theory, it is impossible to identify the final number of participants in advance [34,55-57]. Participants are selected on the basis of theoretical sampling to fill gaps in the data until saturation is achieved. If saturation is not reached, additional participants will be recruited.

### Data analysis

We will use the same analytic processes for constant comparison and situational analysis as described for Study 1. However, we will take the coding to higher levels of abstraction and expand the analysis of the relationships among the codes and categories to produce a grounded theory that has “fit, work, and grab”. That is, it fits the data (which can be verified by an outsider pursuing the researchers’ audit trail); it works to explain the situation under study (which would be verified by returning the results to participants for their review and corroboration); and it has “grab” – that is, it intuitively makes sense to the reader, has a ring of “truth” and often leads to a “but of course” or an “ah ha” reaction by participants.

## Discussion

Foundations of this research are theoretical perspectives on complex adaptive systems, critical social justice, and intersectionality. The combination of complexity thinking with critical social justice and intersectionality is particularly relevant to this research because it provides a framework for theorizing about both the institutional structures and multiple social processes that impact the development of health inequities at multiple levels.

### Complex adaptive systems

Public health systems can be seen as complex adaptive systems. A complex adaptive system is made up of many parts and has the capacity to adapt to a changing environment. The focus is on interactions and relationships, recognizing that multiple interacting actors may create emergent properties at the systems level [23]. Context is foregrounded and there is acknowledgement of the mutual adaptation of interventions and their environments. A complex intervention, such as a change in public health policy, is inserted into a complex system [41], where it interacts with agents in the system to produce particular outcomes that, in turn, lead to changes in the environment. The interactions do not proceed in a linear fashion and interventions change as they are implemented through interaction with the systems surrounding them. Context is, in fact, part of the intervention as it unfolds and this interaction provides a rich opportunity for learning.

Complexity science concepts are increasingly being used in the health and social sciences [58-63] and in public and population health [64-67]. Despite the emerging consensus on the promise of a complexity approach, this “complexity turn” has not gone unchallenged, and various critics have argued that complexity science concepts may be used inconsistently with the origins of complexity science [63,68-71]. The literature is just beginning to emerge on the methodological and design implications of complexity concepts for studying complex systems including population health interventions [40,72,73]. This program of research will contribute to that knowledge.

### Critical social justice

Critical perspectives in social justice emphasize the structural forces that shape health inequities. Current work in the fields of public health and feminist ethics are important to illuminate power, political structures, and social processes in the production of health inequities. The intersection of structural forces contributes to the creation of systemic conditions that produce social suffering and health inequities [74-76]. People, by virtue of their social position, are situated within historical, political and social contexts and consequent social positions impact their health. Feminist scholars such as Young, [77] and Fraser[78] highlight the importance of social structures, institutional context and political processes as important factors that shape the distribution of social goods. Fraser differentiates between who is represented in policy making and who is recognized as suffering injustices, bringing to bear the importance of recognizing intersecting vulnerabilities associated with age, gender, ethnicity and democracy in policy processes.

In public health ethics, Powers and Faden [79], argue for attention to the health of the whole population with specific attention to the health of disadvantaged groups within the population. They clearly articulate six key dimensions of well-being that individuals should be able to access. However, the focus is not on judging whether individuals have a certain level of well-being but rather the degree to which social systems and the social conditions allow for sufficiency or well-being in all these dimensions. In articulating fundamental values of public health ethics, Baylis et al.[80] and Kenny et al.[81] promote relational solidarity as a core principle. Relational solidarity is an expressed commitment to acknowledging important differences between people, recognizing disadvantages, systemic discrimination and power differentials among groups. Central to this view, is the successful dismantling of systems of privilege located in social structures and public policy.

### Intersectionality

Intersectionality is an approach to considering age, gender, ethnicity, class, ability, sexual identity and other dimensions of difference that intersect and contribute to the production of health inequities. That is, multiple types of discrimination or disadvantage may combine and interact to produce social inequity in ways that are not obvious from looking at each type of disadvantage on its own. Intersectionality is one way of understanding the pathways that influence the development of health inequities [82-84]. Walby [84] proposes that intersectionality is compatible with complexity thinking and that complexity theory can be extended to include intersectional perspectives at the systems level. She argues for consideration of both social relations (regimes) and institutional structures (domains) that operate to produce inequities at multiple levels (individual, group and system).

This research program addresses fundamental concerns that are central to reducing health inequities. First, the degree to which health inequities reduction has been embraced as a priority within the health sector is not clear. Second, there is limited information about the extent to which intersectoral collaboration has been taken up to reduce health inequities despite frequent calls for this sort of collaboration. Third, there is a proliferation of health equity tools in the literature that have not been subjected to a critical analysis and there is little practical guidance to support use of such tools. Lastly, health inequities reduction, including the application of an equity lens, engages with fundamental issues of power at many levels and this research seeks to expand knowledge of ethical issues and the development of public health ethics frameworks. The research is methodologically innovative and will provide new knowledge of methods for studying complex public health interventions. It will also expand our thinking on integrating complexity and multiple intersecting social systems perspectives on population health intervention research.

## Competing interests

There are no competing interests.

## Authors’ contributions

BP made substantial intellectual contributions to the conceptualization of this program of study, writing the original grant, the preparation of this manuscript and final approval of the version to be published. MM made substantial intellectual contributions to the conceptualization of this program of study, writing the original grant, the preparation of this manuscript, and final approval of the version to be published. TH made substantial intellectual contributions to the conceptualization of this program of study, editing and approval of the final manuscript. WM contributed to writing the original grant for this study and to the content and preparation of this manuscript. KP contributed to the preparation of the manuscript, critical revisions and editing. All authors read and approved the final manuscript.

## Pre-publication history

The pre-publication history for this paper can be accessed here:

http://www.biomedcentral.com/1471-2458/13/550/prepub
